# Mutation Analysis of ASXL1 in Normal Karyotype Myelodysplastic Syndromes: Experience From Pakistan

**DOI:** 10.1002/cnr2.70078

**Published:** 2024-12-29

**Authors:** Sumera Shaikh, Nida Anwar, Saba Shahid, Shamim Mushtaq, Naveena Fatima, Saima Siddiqui, Qammar Jammal

**Affiliations:** ^1^ Department of Hematology King Faisal Hospital Makkah Kingdom of Saudi Arabia; ^2^ Department of Clinical Hematology National Institute of Blood Diseases and Bone Marrow Transplantation Karachi Pakistan; ^3^ Department of Molecular Diagnostics Al Qassimi Hospital Sharjah UAE; ^4^ Deaprtment of Biochemistry Ziauddin University Karachi Pakistan; ^5^ Department of Research and Clinical trails Metrics Research Pvt. Ltd Karachi Pakistan

**Keywords:** ASXL1 mutation, myelodysplastic syndromes, normal karyotype

## Abstract

**Background:**

The challenges in advancing treatment modalities for myelodysplastic syndromes (MDS) may stem from an incomplete understanding of the disease's complex pathophysiology and lack of reliable prognostic molecular markers. Advanced genomic techniques have revolutionized disease prognosis and risk stratification, particularly for cases with a normal karyotype.

**Aim:**

The present study aimed to analyze ASXL1 mutations in MDS exhibiting a normal karyotype.

**Methods and Results:**

The study enrolled 41 MDS patients with normal karyotypes. Genomic DNA was extracted from peripheral blood samples, followed by Sanger sequencing. The Chi‐square and Mann–Whitney *U* tests were applied to assess the association of age and hemogram with the occurrence of mutations. Survival analysis was done via the Kaplan–Meier method. Statistical operations were performed employing the IBM SPSS Statistics version 24. The patients had a median age of 40 years comprising 28 (68.3%) males and 13 (31.7%) females. ASXL1 mutations were identified in 8 (19.5%), particularly stopgain single nucleotide variant (SNV) and frameshift insertion. The median total leucocyte count × 10^9^/L and blast percentage among the patients with ASXL1 mutation were 3.9 and 4.5, respectively. A survival of 85 weeks was recorded for ASXL1‐mutated and nonmutated cases. The association of ASXL1 mutation status with age and studied hematological parameters was found nonsignificant.

**Conclusion:**

ASXL1 mutation plays a pivotal role in MDS prognosis, warranting assessment at diagnosis for risk stratification and treatment decisions. Early identification of ASXL1 mutation, particularly in low‐risk patients or those with normal karyotype, is imminent to identify patients exhibiting unfavorable prognosis. Integrating molecular insights into existing risk assessment holds significance for improved clinical outcomes, reduction in disease progression, and ultimately the improved quality of life and should be identified early in the course of disease even in the developing world.

## 1 Background

Myelodysplastic syndromes (MDS) are a heterogeneous group of clonal stem cell disorders characterized by peripheral blood cytopenia, morphological dysplasia, and a tendency for evolution to acute myeloid leukemia (AML) [1, 2, 3, 4]. Diagnosis is based on morphological dysplasia in blood and bone marrow with karyotyping having a gold standard value not only for diagnosis but also for the assessment of disease prognosis [1]. The international prognostic scoring system (IPSS‐R) plays a crucial role in anticipating disease outcomes and estimation of disease progression. Nevertheless, the utilization of mutational profiles being an essential preliminary evaluation tool has revolutionized the current molecular prognostic scoring system (IPSS‐M) [2].

Chromosomal analysis by karyotyping and several other techniques such as fluorescence in situ hybridization (FISH), chromosome banding analysis (CBA), genomic microarrays, and genome mapping by next‐generation gene sequencing has now created a paradigm shift in the prognosis assessment [4]. Therefore, the newer IPSS‐M incorporates mutational analysis to stratify and revise treatment strategies according to the disease risk [2]. Around half of MDS cases having a normal karyotype are stratified as standard or intermediate risk as per IPSS‐R [2]. However, when molecular analysis is incorporated; disease risk could be revised as high risk if adverse risk molecular mutation is identified [2, 5]. Thus, the use of genomic sequencing has improved the assessment of actual normal karyotype instances by 20%–30% [4, 6].

Next‐generation sequencing (NGS) and Sanger sequencing are now playing a pivotal role in the paradigm shift of identifying disease risk and improving the diagnostic approach to alter disease management in predicting transformation to AML early in the disease course, with the extraordinary potential of gene sequencing, especially when cytogenetic markers do not establish clonality [1, 2]. Mutation in one of several genes such as TP53, EZH2, ETV6, RUNX1, and ASXL1 is indicative of poor prognosis for MDS while SF3B1 mutations have a better clinical outcome in 10.8% of de novo AML, 8% myeloproliferative neoplasia, 49% of chronic myelomonocytic leukemia, and 23% of post‐MDS AML. One of the national studies reported 54.5% of patients with normal karyotype exhibiting ASXL‐1 mutation via NGS in 9.09% [1].

The mechanistic link between *ASXL1* mutations and clonal hematopoiesis (CH) involves the epigenetic regulator ASXL1, which is often mutated in CH and various myeloid neoplasms. ASXL1 mutations may contribute to pathogenesis through gain‐of‐function, dominant‐negative or loss‐of‐function mutations. Unique histone modifications, autoimmunity, and signal transduction may be accountable for ASXL1 mutation‐induced CH [7]. A number of ASXL1 mutations have been found in the literature. Most ASXL1 mutations in MDS are frameshift or nonsense mutations that result in a truncated protein, especially in the C‐terminal region of the gene. According to various genomic studies and databases, over 100 identified mutations in the ASXL1 gene have been linked to various myeloid malignancies, particularly MDS, and some of these are more commonly associated with MDS than with other hematologic malignancies, resulting in loss of function, dysregulation of transcriptional repression, and chromatin remodeling [8, 9, 10, 11, 12].

Some of the most common ASXL1 mutations in MDS include p.G646WfsX12, which results in a truncated ASXL1 protein that lacks the PHD finger domain. The other mutation is p.R693X, which is a nonsense mutation that causes a premature stop codon and truncation. Other notable mutations include p.E635fs and p.R404X, which are commonly seen in MDS cases with a poor prognosis. Meta‐analyses have also shown that MDS with ASXL1 mutations have a significantly lower overall survival (OS) and a higher likelihood of progression to AML, making them an important factor in prognosis and therapy [8, 9, 10, 11, 12, 13, 14, 15].

Significant challenges in developing treatment strategies for MDS in our part of the world could be attributable to an incomplete comprehension of the disease's complex pathophysiology and the absence of dependable prognostic molecular markers. In MDS, ASXL1 mutation is found with a frequency of 20.7%. Heterozygous frameshift mutations (70%) are more prevalent than heterozygous missense mutations. The analysis of ASXL1 mutational status should be incorporated into the genetic assessment of MDS patients, as the mutation status is now integrated into molecular scoring systems being mandatory in the Western world [2].

Pakistan is a low‐middle‐income country (LMIC) where health and research are limited, and diagnosis and treatment of MDS are quite challenging contrary to the developed world where genetics and genomics are now considered to be instrumental for improving risk stratification, diagnosis, treatment, and prevention [1]. Identification of ASXl‐1 mutation in MDS at least in those patients carrying low risk as per IPSS‐R could help identify the group of high‐risk characteristics incorporating ASXL1 mutation and thus could be treated accordingly by proceeding early to allogenic stem cell transplant (allo SCT).

Thus, in this study, the efforts were mainly focused on the MDS patient exhibiting normal karyotype. Mutations in *ASXL1* have previously been linked to the absence of cytogenetic abnormalities associated with MDS and intermediate cytogenetic risk in patients with AML‐MRC [5]. IPSS‐M has been incorporated into newer risk assessment strategies for expanded information in the prognosis of MDS pathogenesis which largely affects the clinical practice and thus may be helpful for better survival and prediction of median time for disease transformation. ASXL1, being one of the significant mutations associated independently with adverse outcomes and early disease transformation, should be assessed at baseline at least where a robust NGS panel is not accessible to identify this somatic mutation in MDS to better predict the clinical outcome in patients exhibiting normal karyotype. Thus, the present work aimed at identifying ASXl‐1 mutation by Sanger sequencing.

## 2 Methods

This was an analytical cross‐sectional study. Patients were enrolled from December 2022 to December 2023 at the National Institute of Blood Diseases and Bone Marrow Transplant (NIBD & BMT) Karachi, Pakistan. The research protocol received approval from the Research Ethics Committee of NIBD adhering to the principles of the Declaration of Helsinki. Written informed consent for the study and publication of its outcomes was taken from study subjects. Until DNA isolation and subsequent analysis, all recruited patients' peripheral venous blood samples were collected in EDTA tubes and stored at 4°C. The study included de novo diagnosed MDS having normal karyotype on cytogenetic analysis. Patients having a history of previous chemotherapy or radiotherapy or de novo MDS but having karyotype abnormality were excluded from the study. The association of hemogram variables with the presence of ASXL1 mutation was analyzed as an outcome of the study. Association of survival events, gender, and MDS category with the presence of ASXL1 mutation were also evaluated.

### 2.1 DNA Extraction

Peripheral blood genomic DNA was extracted employing the QIAamp DNA Blood Mini Kit (Qiagen, Hilden, Germany), following the manufacturer's instructions. The extracted DNA's quality was evaluated using a 2% agarose gel and quantified using the Qubit DNA HS Assay Kit (Invitrogen, Thermo Fisher Scientific, USA).

### 2.2 Mutation Screening

The polymerase chain reaction (PCR) amplification of all 13 exons of *ASXL1* was carried out as described by Nie Y et al. in 2018, combining 25 μL of Dream Taq Hot Start Green PCR Master Mix (2X; cat. no. K9021; Thermo Fisher Scientific Inc., Waltham, MA, USA), along with 0.8 μM of both forward and reverse primers, 200 ng of template DNA, and nuclease‐free water to achieve a final volume of 50 μL. The PCR was conducted with an initial denaturation step at 95°C for 3–5 min, followed by 35 cycles at 94°C for 30 s, 55°C for 30 s, and 72°C for 1 min. A final extension step at 72°C for 5 min for final extension step. The assessment of the quality of the amplified exons of the *ASXL1* was conducted using a 2% agarose gel, and its quantification was performed using the Qubit DNA HS Assay Kit (Invitrogen, Thermo Fisher Scientific, USA). Subsequently, purification of amplified products was carried out by ExoSAP‐IT (Applied Biosystem, Thermo Fisher Scientific) kit to remove the remaining primers and other impurities.

The PCR products were bidirectional sequenced by the Big Dye Terminator v1.1 Cycle Sequencing Reaction kit (Applied Biosystems, Foster City, CA) according to the manufacturer's protocol using an ABI PRISM 3500 DNA Analyzer (Applied Biosystems). Sequence electropherograms were analyzed using Applied Biosystems software and reviewed manually. The analysis with identified gene alterations was sourced from the Ensembl Genome Browser (http://www.ensembl.org). Known single nucleotide polymorphisms were discerned by referencing the NCBI dbSNP databases.

### 2.3 Statistical Analysis

The data were recorded and analyzed by using the Statistical Package for Social Sciences (SPSS) version 24. The Shapiro–Wilk test was applied, and data were not found normally distributed. Median and interquartile range (IQR) were computed for quantitative variables while frequency and percentages were calculated for qualitative variables. Mann–Whitney *U* test was applied to assess the mean difference of age and hemogram between the groups and Chi‐square and Fisher's exact tests were used to observe the association of gender, survival events, and MDS category with the occurrence of mutation. The *p*‐value of < 0.05 was significant. The survival analysis was done via the Kaplan–Meier method.

## 3 Results

**FIGURE 1:**
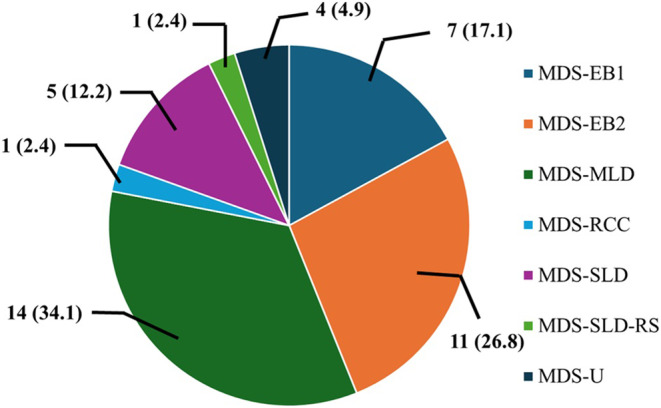
The pie chart shows the distribution of myelodysplastic syndrome subtypes within the studied cohort. The parenthesis reflects the associated percentages with each class. Myelodysplastic syndrome with excess blasts 1(MDS‐EB1), myelodysplastic syndrome with excess blasts 2 (MDS‐EB2), myelodysplastic syndrome with multilineage dysplasia (MDS‐MLD), myelodysplastic syndrome with refractory cytopenia of childhood (MDS‐RCC), myelodysplastic syndrome with single lineage dysplasia (MDS‐SLD), myelodysplastic syndrome single lineage dysplasia with ring sideroblasts (MDS‐SLD‐RS), and myelodysplastic syndrome unclassified (MDS‐U).

The study included 41 patients diagnosed with MDS having male predominance, that is, 28 (68.3%) whereas females comprised 13 (31.7%). The median age of the patients was 40 years. The distribution of MDS exhibited variable trends across different categories. In particular, the myelodysplastic syndrome with multilineage dysplasia (MDS‐MLD) emerged as the most prevalent subtype accounting for 34.1% of cases. It was trailed by myelodysplastic syndrome with excess blasts 2 (MDS‐EB2) with frequency of 26.8%. Additionally, a subset characterized by single lineage dysplasia was also notable in some participants as presented in Figure 1. The descriptive statistics of the study participants are presented in Table 1.

**TABLE 1:** The overall characteristics of studied variables among the MDS patients.

Variable	Descriptive statistics
Gender	
Male, *N* (%)	28 (68.3)
Female, *N* (%)	13 (31.7)
Age in years [median (IQR)]	40 (30)
ASXL1 mutations	
Yes	08 (19.5)
No	33 (80.5)
Hemogram	
Hemoglobin in g/dL [median (IQR)]	8.5 (2.2)
Total leucocyte count × 10^9^/L [median (IQR)]	3.7 (5.1)
Absolute neutrophil count [median (IQR)]	1.5 (1.1)
Platelet × 10^9^/L [median (IQR)]	73 (53)
Blast in % age [median (IQR)]	04 (09)

Intriguingly, when compared among *ASXL1* mutation‐positive and the counterpart cases, no significant distinctions in key parameters of the hemogram i.e., levels of hemoglobin, neutrophil count, leukocyte count, and blast percentage were observed (Table 2). This highlights that the presence of the studied mutation may not exert a notable influence on these clinical parameters in our cohort. Furthermore, the dissection within MDS categories also did not reveal any significant associations among the mutation status and distribution of disease subtype, thereby suggesting no direct influence (Table 2).

**TABLE 2:** Association of study variables with the presence of *ASXL1* mutation.

Variables	ASXL1 mutation		*p*
	Yes	No	
Median age	56	39	0.292
Median hemoglobin (g/dL)	8.7	8.5	0.669
Median total leucocyte count × 10^9^/L	3.9	3.4	0.657
Median absolute neutrophil count	1.8	1.5	0.647
Median platelet × 10^9^/L	71	73	0.633
Median blast in % age	4.5	4	0.745
Myelodysplastic syndrome with excess blasts 1 (MDS‐EB1)	2	5	0.351^a^
Myelodysplastic syndrome with excess blasts 2 (MDS‐EB2)	1	10	
Myelodysplastic syndrome with multilineage dysplasia (MDS‐MLD)	4	10	
Myelodysplastic syndrome with refractory cytopenia of childhood (MDS‐RCC)	0	1	
Myelodysplastic syndrome with single lineage dysplasia MDS‐SLD	0	5	
Myelodysplastic syndrome single lineage dysplasia with ring sideroblasts (MDS‐SLD‐RS)	1	0	
Myelodysplastic syndrome unclassified (MDS‐U)	0	2	
Male	7	21	0.398^b^
Female	1	12	
Alive	4	14	0.693^b^
Expire	3	16	

^a^ Chi‐square test.

^b^ Fisher's exact test.

Mutations of *ASXL1* in the studied cohort are presented in Table 3 with the identification of *ASXL1* mutations in eight (19.5%) patients. The majority of mutations were stopgain SNV followed by frameshift insertion. One of the nonsynonymous SNVs found in one of the patients has no clinical significance and hence was not included in the statistical analysis. Germline stopgain SNVs (c.C1894T; p.R693X, c.C2077T; p.R693X, c.C2932TX; p.Q978, c.C3115T; p.Q1039X) were detected in two patients. Somatic frameshift insertions (c.1744dupG; p. G581fs, c.1927dupG; p. G642fs, c.2889dupA; p. T963fs, c.3072dupA; p. T1024fs) were found in two patients, while the variants in the remaining patients were rare, with variant allele frequencies (VAFs) less than 0.001% including two stopgain (rs373221034, rs1221031683) and one frameshift insertion. Mutations c.C2077T; p.R693X, c.1927dupG; p. G642fs, and c.C3115T; p.Q1039X are only documented in the literature.

**TABLE 3:** Mutations identified in *ASXL1* in MDS patients.

Patients	Variant class	Nucleotide changes Exon 11		Amino acid changes Exon 12	
3	stopgain_SNV	c.C1894T	p.R693X	c.C2077T	p.R632X
4	frameshift_insertion	c.1744dupG	p.G581fs	c.1927dupG	p.G642fs
	stopgain_SNV	c.C1894T	p.R632X	c.C2077T	p.R693X
8	stopgain_SNV	c.C2932T	p.Q978X	c.C3115T	p.Q1039X
11	stopgain_SNV	c.C1894T	p.R632X	c.C2077T	p.R693X
16	frameshift_insertion	c.2889dupA	p.T963fs	c.3072dupA	p.T1024fs
18	stopgain_SNV	c.C2932TX	p.Q978	c.C3115T	p.Q1039X
22	synonymous_SNV	c.C2487T	p.L829L	c.C2670T	p.L890L
BMS27.1	non synonymous_SNV^a^			c.G4456A	p. A1486T

Abbreviation: SNV, single nucleotide variant.

^a^ No clinical significance.

**FIGURE 2:**
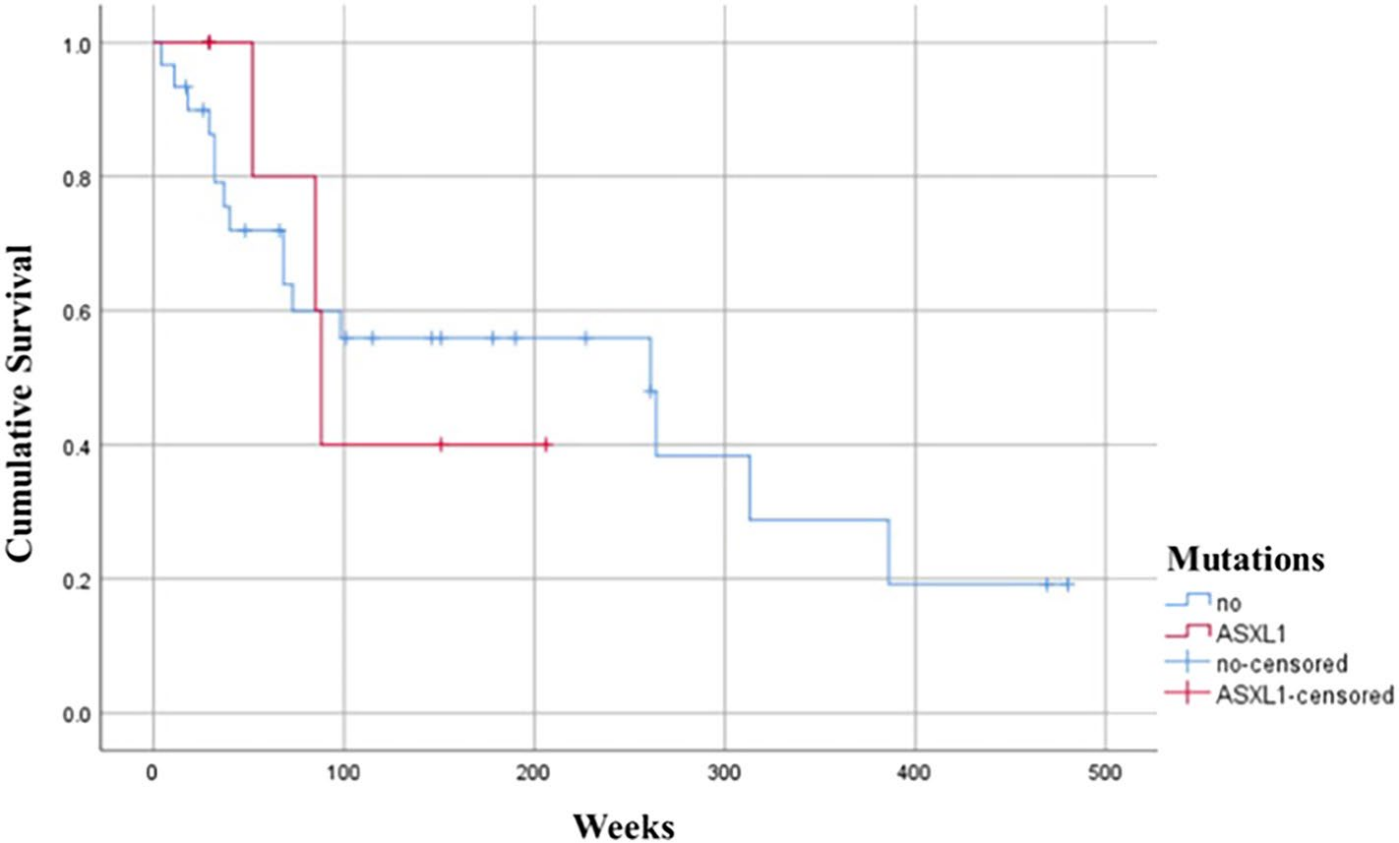
Kaplan–Meier survival curve for *ASXL1* mutation‐positive patients.

Survival analysis for the available data (*n* = 37) demonstrated a survival rate of 57% among ASXL1 mutation‐positive patients compared to the negative cases featuring a value of 46.7% as depicted in Figure 2. However, the differences observed were statistically nonsignificant (Table 4).

**TABLE 4:** A comparison of survival distribution of *ASXL1* mutation positive and negative cases.

ASXL1 Mutation	Survival analysis				Log rank (Mantel‐Cox)
	median (weeks)	IQR	95% Confidence interval		
			Lower bound	Upper bound	
Yes	85	122	81.559	94.441	0.892
No	85	204	55.779	466.221	

## 4 Discussion

*ASXL1* mutations are noticeable among the observed genetic abnormalities in MDS. The majority, around 80%–90%, of mutations are linked to the disease‐impact genes responsible for epigenetic regulation and splicing, crucial for gene expression and cell division control [1]. With the prevalence of these variations and cytogenetic anomalies, genomic instability is believed to be pivotal in the pathogenesis of disease [16].

In this study, we aimed to identify *ASXL1* mutation in patients having normal karyotypes and analyze their clinical and genetic characteristics. Our findings included the demographic trend, distribution of subtypes, mutation analysis, and their potential associations with disease prognosis and survival. There was male predominance comprising 68.3% of patients. Gender represents a significant factor contributing to disease diversity, the underlying pathophysiological mechanisms, clinical manifestations, and disease prognosis. Thus, recognizing the significance of gender in clinical studies, and adopting a sex‐informed approach is an emerging standard in precision medicine [17, 18, 19, 20, 21]. The median age, however, recorded in these studies was higher which contrasts with the present finding. Furthermore, the occurrence of MDS is reported to rise exponentially after 60 years of age [20, 22]. The median age of 40 years herein underscores the potential impact of disease, warranting intensified and curative treatment approaches like allo SCT.

The diagnosis of MDS is challenging specifically in patients with minimal dysplasia and pancytopenia, and is subject to considerable variability among observers, with approximate disagreement rates of 30–40 in diagnosis [23]. This difficulty is further heightened for patients exhibiting normal karyotypes or lacking the typical dysplastic features in the bone marrow. Other complexities may arise from unsuccessful biopsies, often those resulting from extensive myelofibrosis and marked hypocellularity [24, 25]. This is highlighted in the research conducted by Bersanelli and coworkers, who analyzed 2043 MDS patients from the EuroMDS consortium, and 1195 (59%) patients harbored normal karyotypes [4].

The World Health Organization (WHO) proposed and modified the classification system based on the presence of specific morphological and cytogenetic features, aimed at better characterization of diagnosis and the management [26, 27]. In our study, MDS‐MLD was identified as the most prevalent subtype, representing 34.1% of cases followed by MDS‐EB2 (26.8%), highlighting individuals with an excess of blasts in bone marrow. Similar observations were made by Wada and coworkers who reported the presence of MDS with multilineage dysplasia in most of the cases (23.2%). Furthermore, MDS‐EB1 and MDS‐SLD, which represent subsets with single lineage dysplasia, were also observed. Consistent with these findings, Lee and colleagues reported that the majority of the MDS patients (~52.8%) featured excess blasts [21]. In contrast with the present study, MDS cases with single and multilineage dysplasia alongside ring sideroblasts were observed in 6.4% and 3.8% of the studied patients [21]. The varied distribution is attributed to the intrinsic heterogeneous nature of MDS, characterized by differing levels of blast proliferation and dysplasia across distinct subtypes. The proper understanding and accurate diagnosis by incorporating molecular assessment enables better treatment strategies and prognostic evaluations [2].

Genomic instability is a determining factor in the development of neoplastic disease, and mutations in *ASXL1* are prominent among the observed variants in MDS [16]. The *ASXL1* gene located on chromosome band 20q1 encodes a highly conserved protein belonging to the enhancer of trithorax and polycomb (ETP) family of genes [28]. In our study, we observed 19.5% of individuals exhibiting ASXL*1*, including stopgain SNV and frameshift insertions. This frequency is somewhat similar to the findings reported by Chen and colleagues. In their extensive review of 466 MDS patients, *ASXL1* mutations were identified in 106 patients (22.7%), including frameshift and nonsense mutations. In their study, patients with refractory anemia (RA) and refractory anemia with ringed sideroblasts (RARS) had lower incidence of *ASXL1* mutation compared to those with patients exhibiting excess blasts (MDS‐EB) which is contrary to our findings where we found a similar median blast count in patients with and without *ASXL1* mutation [29, 30, 31]. Overall, their study suggests that *ASXL1* mutation along with other genetic alterations may play a role in the development of MDS but have a limited influence on disease progression.

The *ASXL1* mutation p.R693X, induces a premature termination codon, anticipated to lead to either truncation of the encoded protein or its absence via nonsense‐mediated decay. This truncated mutation has been widely reported to be associated with poor prognosis in myeloid malignancies which was found in three of our patients [32]. In patient 4 (p. G642fs), a frameshift insertion was found. Similar *ASXL1* mutations were identified by Wang and colleagues as recurrent somatic events in familial MDS.

Metzeler and colleagues analyzed 423 adult patients for *ASXL1* mutation and found out that it was more common in older patients (> 60 years) and associated with unfavorable outcomes [6]. However, we did not find any significant association of *ASXL1* mutation with age in our cohort. One of the possible reasons could be the younger median age of MDS in our part of the world [1]. Their study was one of the first studies assessing primary *ASXL1* mutation in karyotype normal AML demonstrating *ASXL1* mutated older patients with ELN favorable group having unfavorable outcomes by virtue of the mutation warranting early intensified treatment approaches and upfront consideration for transplant [6].

Gelsi‐Boyer and colleagues found that *ASXL1* mutations affect the 12th exon of the gene in the majority, and this was a similar finding in our study [31]. They also highlighted that *ASXL1* mutations are usually heterozygous, suggesting haploinsufficiency as a key pathogenic factor. However, a truncated *ASXL1* protein could also have a dominant‐negative role in titrating out interacting protein indicating these mutations as loss‐of‐function disease alleles. *ASXL1* usually constitutes an early hit in leukemogenesis preceding other mutations like *TET2* and *JAK2* mutations. However, our study exclusively focused on identifying *ASXL1* mutations using Sanger sequencing, without assessing other gene mutations. Additionally, their study found the potential loss or acquisition of mutations during relapse. However, our mutation analysis was conducted solely at baseline, without assessing mutation status during disease transformation [31].

Thol and colleagues analyzed the prognostic effect of *ASXL1* mutation, analyzing point and frameshift mutations separately. They observed that frameshift mutations had an independent prognostic effect in MDS patients, unlike point mutations. However, our study did not detect any point mutations, and only two patients had frameshift mutations (p.G581fs, p.G642fs, p.T963fs, p.T1024fs). Thus, a larger sample size and future prospective studies in our part of the world are needed to further validate this observation [32].

Another study by Sallman and colleagues analyzing mutations in MDS depicted that *TP53, EZH2, ETV6, RUNX1*, and *ASXL1* genes are associated with inferior survival, although *ASXL1* mutations showed only borderline significance. This observation is somewhat similar to our findings, as we did not find any significant adverse implication of this mutation in our cohort. However, the small sample size of our study limits the generalization of this observation. Furthermore, their research encompassed chronic myelomonocytic leukemia (CMML) patients in addition to MDS and concluded that *ASXL1* mutation was predictive for outcomes in CMML but did not affect outcomes in MDS, even when considering missense mutations [33].

Our study assessed the clinical and mutational characteristics of *ASXL1* mutations in karyotype‐negative MDS. So far, this mutation has been reported to be independently associated with unfavorable disease outcomes warranting early identification for better stratification of disease risk and treatment. NGS is not widely accessible for molecular characterization of risk assessment in the developing world and thus Sanger sequencing could be implemented as a relatively cost‐effective strategy to identify high‐risk mutations warranting treatment modifications. Despite the challenges encountered in our region, our goal was to analyze this high‐risk mutation and assess its implications within our cohort.

Our study had a few limitations, including a small sample size and selection bias, and because it was a cross‐sectional study, no causal association could be concluded. However, additional longitudinal research with larger sample sizes is required in the future to build robust inferences from joint national multicenter analysis. Furthermore, research of additional mutations in targeted therapy for ASXL1‐positive MDS with long‐term follow‐up may yield further evidence to add or update prognostic inferences in our region of the world. Nonetheless, our work is among the first in the country to report ASXL1 outcomes in normal karyotype MDS.

## 5 Conclusion

Our findings highlighted the various clinical characteristics in terms of MDS subtypes, hematological parameters, and various *ASXL1* mutations in karyotype‐negative patients. Though no significant associations were found among *ASXL1* mutated and nonmutated subgroups in terms of clinical parameters and survival, it still demands further exploration with a larger sample size and multicenter collaborative studies to highlight the mutational profile and outcome implications in our part of the world. Overall, the results still provide valuable insights, highlighting the importance of customized mutation panels to plan revised and risk‐stratified treatment approaches, particularly for high‐risk driver mutations like *ASXL1*.

## Author Contributions

**Sumera Shaikh:** writing – original draft. **Nida Anwar:** conceptualization, methodology, data curation, supervision, project administration, writing – original draft, writing – review and editing, investigation, validation. **Saba Shahid:** methodology, software, data curation, writing – review and editing, writing – original draft. **Shamim Mushtaq:** writing – review and editing. **Naveena Fatima:** formal analysis, writing – original draft, writing – review and editing, data curation. **Saima Siddiqui:** writing – review and editing. **Qammar Jammal:** writing – review and editing.

## Ethics Statement

The research protocol received approval from the Research Ethics Committee of NIBD adhering to the principles of the Declaration of Helsinki. Written informed consent for the study and publication of its outcomes was taken from study subjects.

## Consent

The authors have nothing to report.

## Conflicts of Interest

The authors declare no conflicts of interest.

## Data Availability

The data that support the findings of this study are available on request from the corresponding author. The data are not publicly available due to privacy or ethical restrictions.
